# Hyperbaric Oxygen Increases Stem Cell Proliferation, Angiogenesis and Wound-Healing Ability of WJ-MSCs in Diabetic Mice

**DOI:** 10.3389/fphys.2018.00995

**Published:** 2018-07-30

**Authors:** Isaac Peña-Villalobos, Ignacio Casanova-Maldonado, Pablo Lois, Catalina Prieto, Carolina Pizarro, José Lattus, Germán Osorio, Verónica Palma

**Affiliations:** ^1^Laboratorio de Ecofisiología Animal, Departamento de Ecología, Universidad de Chile, Santiago, Chile; ^2^Laboratorio de Células Troncales y Biología del Desarrollo, Departamento de Biología, Universidad de Chile, Santiago, Chile; ^3^Campus Oriente, Department of Obstetrics and Gynecology, Faculty of Medicine, University of Chile, Santiago, Chile; ^4^Osorio Hermanos & Cia. Ltd., Quillota, Chile

**Keywords:** CAM assay, diabetes, HBOT, intestinal stem cells, mesenchymal stem cells

## Abstract

Hyperbaric oxygen therapy (HBOT) is effective for the medical treatment of diverse diseases, infections, and tissue injury. In fact, in recent years there is growing evidence on the beneficial effect of HBOT on non-healing ischemic wounds. However, there is still yet discussion on how this treatment could benefit from combination with regenerative medicine strategies. Here we analyzed the effects of HBOT on three specific aspects of tissue growth, maintenance, and regeneration: (i) modulation of adult rodent (*Mus musculus*) intestinal stem cell turnover rates; (ii) angiogenesis dynamics during the development of the chorio-allantoic membrane (CAM) in *Gallus gallus* embryos; (iii) and wound-healing in a spontaneous type II diabetic mouse model with a low capacity to regenerate skin. To analyze these aspects of tissue growth, maintenance, and regeneration, we used HBOT alone or in combination with cellular therapy. Specifically, Wharton Jelly Mesenchymal Stem cells (WJ-MSC) were embedded in a commercial collagen-scaffold. HBOT did not affect the metabolic rate of adult mice nor of chicken embryos. Notwithstanding, HBOT modified the proliferation rate of stem cells in the mice small intestinal crypts, increased angiogenesis in the CAM, and improved wound-healing and tissue repair in diabetic mice. Moreover, our study demonstrates that combining stem cell therapy and HBOT has a collaborative effect on wound-healing. In summary, our data underscore the importance of oxygen tension as a regulator of stem cell biology and support the potential use of oxygenation in clinical treatments.

## Introduction

Hyperbaric oxygen therapy (HBOT) is an effective medical treatment that can be used in the therapy of diverse diseases, infections, and tissue injury. Specifically, this treatment consists of increasing the oxygen tension in tissues close to 400 mm Hg by means of the use of an experimental chamber (Ambiru et al., 2008). HBOT follows Henry’s law, i.e., gas solubility in a solution depends on the partial pressure applied to it, but does not change the affinity of hemoglobin for oxygen.

In clinical applications, HBOT has been shown to have beneficial effects. For example, HBOT has been used for the treatment of carbon monoxide poisoning, necrotizing soft tissue, decompression sickness, and arterial gas embolism. Additionally, the effects on skeletal muscle repair following injury and jawbone osteoradionecrosis have been reported (Nylander et al., 1988; Gregorevic et al., 2000, 2002; Asano et al., 2007; Vidigal et al., 2007). More recently, HBOT has been used for the treatment of ischemia-reperfusion (Chen et al., 1998; Francis, et al., 2017). HBOT is also used as an adjuvant for several other conditions including burns, autism, crush injuries, and compartment syndromes. Professional sports teams have also favored the use of HBOT to speed injury (Strauss et al., 1983; Strauss and Hart, 1984; Skyhar et al., 1986; Cianci and Sato, 1994; Goldfarb et al., 2016). Moreover, several studies have proposed that HBOT mobilizes stem cells by improving homing and subsequent engraftment in injured tissues, which would explain, in part, the therapeutic effects of HBOT (Zádori et al., 2011; van Neck et al., 2017).

Despite all of the evidence of HBOT’s positive effects, its clinical value is still yet under discussion (see Dulai et al., 2014; An et al., 2015; Fox et al., 2015; Goldfarb et al., 2016; Chong and Rice, 2016; Borab et al., 2017). One difficulty of assessing the positive effects of HBOT in both experimental and clinical use includes the lack of standardized treatment protocols. Specifically, there is no clearly defined amount and duration of HBOT sessions, and the pressure conditions applied during sessions often vary greatly. **Table 1** shows the variability among HBOT protocols. Furthermore, significant progress has been made in recent years in our understanding of the mechanisms by which HBOT affects cells and tissues, but many unanswered questions remain. In particular, the physiological effects of HBOT on stem cell dynamics, metabolism, and tissue repair have been poorly documented.

**Table 1:** Summary of the different HBO treatments used worldwide since 2001.

Organism	Pressure (ATA)	Session duration (h)	Sessions	Reference
Rat	2	1	1	Lin and Wan, 2008
	2.8	1	1	Yang et al., 2010
	2.5	2	5	Takeyama et al., 2007
	2.8	0.75	2/day, 1 day	Palzur et al., 2008
	3	2	5 days, 4 weeks	Kurt et al., 2008
	2.5	1	2/day, 3 days	Liu et al., 2008
	∼6	0.08	1	Li et al., 2008
	3	1	2/day, 28 days	Gregorevic et al., 2001
	2	1	1	Imperatore et al., 2006
	3	1.5	1	Chu et al., 2007
	2	1	7	Wang et al., 2008
Mice	2	1	1/day, 30 days	Dave et al., 2003
	3	1	1/day, 14 days	Asano et al., 2007
	2.5	1	2/day, 3 days	Sakata et al., 2010
	2.5	1.5	1/day, 2 weeks	Chen et al., 2003
	2.5	2	2/day, 5 days	Gajendrareddy et al., 2005
	3	1	1	Veltkamp et al., 2005
	1.5 to 2.4	1	4 days/week	Verma et al., 2015
	2.5	1.5	Three times weekly	Poff et al., 2015
	2	1	4 days	Baratz-Goldstein et al., 2017
	2.5	1.5	1/day, 21 days	Lu et al., 2016
	2.5	1.5	6	Sletta et al., 2017
Rabbit	2.4	1.5	5 days/ 4 weeks	Jan et al., 2009
	2.5	2	20	Atesalp et al., 2009
Human	2	1.5	1/day, 20 days	Boykin and Baylis, 2007
	3	2.08	30 sessions, 5 or 6/week	Hulshof et al., 2002
	2.4	1.5	2/day, 6 days	Kaya et al., 2009
	2	1.5	1/day	Safra et al., 2008
	2	2	10 to 20	Thom et al., 2006
	2.5	0.33	30 sessions, 5/week	Gerlach et al., 2008
	2 to 3	1,5	1/day, 30 days	Duzgun et al., 2008

*The pressure parameters, the duration, and the number of sessions administered to the different vertebrate models are indicated.*

In this study, we analyzed the effects of HBOT on three particular phenomena related with tissue growth, maintenance, and regeneration. (i) We studied the modulation of adult rodent (*Mus musculus*) intestinal stem cell turnover rates. The fast cellular turnover rates of the small intestine (Snippert et al., 2010) have let this organ to be used widely to study stem cell dynamics and the effects of HBOT treatment (Noyer and Brandt, 1999; Buchman et al., 2001; Guven et al., 2009; Rossignol, 2012; Dulai et al., 2014). (ii) We evaluated angiogenesis dynamics during the development of the chorio-allantoic membrane (CAM) in *Gallus gallus* embryos. (iii) Lastly, we studied wound-healing in a spontaneous type II diabetic mouse model with a low capacity to regenerate skin. For this final part of the study, HBOT was administered alone or combined with cellular therapy using Wharton Jelly Mesenchymal Stem cells (WJ-MSC) seeded on commercial scaffolds. Overall, our findings suggest that oxygen acts as a critical regulator of stem cells and highlight the importance of examining these cells and their niches more closely when using HBOT for tissue repair.

## Materials and Methods

In keeping with internal regulations and national requirements, all protocols were approved by the Institutional Animal Care and Use Bioethics Committees of the Faculty of Sciences of the University of Chile and the Bioethics Committee the National Fund for Science and Technology (FONDECYT).

### Hyperbaric Oxygen Therapy

Hyperbaric oxygen therapy was performed in a 19.59 L experimental chamber (Osorio Hermanos & Cia. Ltd.., Quillota, Chile) (**Supplementary Figure S1**). The study animals were positioned either in egg trays (chicken embryos) or individual cages (mice). Then, the remaining air inside the chamber was replaced with 100% O_2_ while the pressure was increased for 15 min until 2 ATA (absolute atmospheres) were reached. The latter conditions were maintained for 1 h. Finally, the decompression from 2 ATA back to atmospheric pressure was done gradually over the course of 15 min. Thus, in total each session took 1.5 h. In parallel with the animals receiving HBOT treatment (from now on referred to as the HBOT group), a control group subjected to normoxia and atmospheric pressure was monitored for the same time. As for the temperature applied during the HBOT sessions, eggs were situated at 38.5°C (±0.1), while mice were treated at 25°C (±0.1). **Table 2** summarizes the different experimental conditions and treatments.

**Table 2:** Summary of experimental designs and respective treatments analyzed in this study.

Experiments	Treatments							
Modulation of stem cell turnover rate in the small intestine of adult rodents (*Mus musculus*)	Control				HBOT			

Development of the CAM in *Gallus gallus* embryos	Control				HBOT			
	Sham	IM	IM + VEGF	IM + WJ-MSC	Sham	IM	IM + VEGF	IM + WJ-MSC

Wound-healing in a spontaneous type II diabetic mouse model (*Mus musculus*)	WT				db/db			
	Sham			HBOT	Sham	HBOT	IM + WJ-MSC	IM + WJ-MSC + HBOT

### Determination of Metabolic Rate in Mice

**FIGURE 1:**
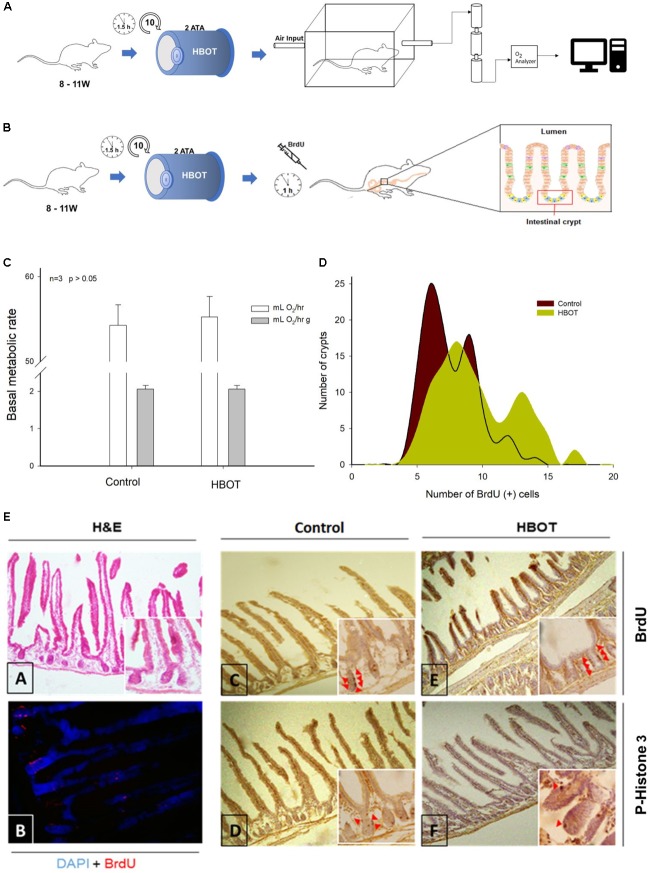
Effects of HBOT on murine intestinal cellular turnover. **(A)** Metabolic assay scheme using adult Balb/c males. Standard flow-through respirometry was used. **(B)** Schematic representation of the cellular turnover of the small intestine of the mice. Here the number of sessions and timing of sessions is included. These sessions involved intraperitoneal injection of BrdU and posterior histological analysis. Bar = 20 μm. **(C)** White bars show basal metabolic rates (mL O_2_/h), which did not differ significantly between the HBOT and control groups. Gray bars show mass specific basal metabolic rates (mL O_2_/h g), evidencing no significant differences between treatments. **(D)** Comparison of distributions of BrdU+ cells per crypts. The brown distribution curve shows four peaks corresponding to BrdU (+) cells per crypt in the control condition while the green distribution curve shows three displaced peaks in the HBOT group (*n* = 3). **(E)** Representative images of BrdU (+) and P-Histone 3 (+) cells found in the intestinal crypts of the control and HBOT groups.

The Basal Metabolic Rate (BMR) of mice was estimated as oxygen consumption ($\dot{V}$O_2_) when the animals were subjected to standard flow-through respirometry conditions (**Figure 1A**). The metabolic measurements were conducted after the last session of HBOT. Mice were weighed and placed in a transparent acrylic chamber (750 mL) located in a temperature controlled (Ta = 30.0 ± 0.5°C) illuminated cabinet (Sable Systems, Henderson, NV, United States). The metabolic chamber received air from a mass flow controller through Bev-A-Line tubing after being dried at 500 mL⋅min-1. The excurrent air passed-through Drierite columns, CO_2_-absorbent Baralyme granules, and Drierite before passing through an O_2_ -analyzer (Turbo Goldfarb model, Sable Systems, Henderson, NV, United States) calibrated with a known mixture of oxygen (20%) and nitrogen (80%), which was certified by chromatography (BOC, Chile). The Turbo Fox mass flow meter was calibrated monthly with a volumetric (bubble) flow meter. The measurement and calibration protocols followed those of Williams and Tieleman (2000). Because water vapor and CO_2_ were scrubbed before entering the O_2_ analyzer, oxygen consumption was calculated as Withers (1977): VO_2_ = [FR × 60 × (Fi O_2_ - O_2_ Fe)]/(1-Fi O_2_), where FR is the flow rate in ml /min STP after correction, and Fi and Fe are the fractional concentrations of O_2_ entering and leaving the metabolic chamber, respectively. The outputs from the oxygen and carbon dioxide analyzer (%) and flow meter were digitalized using Universal Interface II (Sable Systems). The outputs were then recorded on a personal computer using EXPEDATA data acquisition software (Sable Systems). Our sampling interval was 1 s, with measurements performed for 6 h, during animal’s rest phase (08:00 to 14:00 h).

### Proliferation Assay of Rodent Intestinal Tract

#### Intestine Processing

Six adult *Mus musculus* (BALB/c) males were randomly assigned to two groups (control and HBOT) to evaluate stem cell responses in intestinal tissue. Mice were maintained in a 12-h light/dark cycle at 24°C ± 2 and fed with standard laboratory diet (LabDiet, Prolab RMH 3000) and water *ad libitum*.

The HBOT protocol is summarized in **Figure 1B**. Briefly, the rodents were exposed to HBOT conditions in ten group sessions. Before performing the last HBOT session, individuals were injected intraperitoneally with 800 μL of 20 mg/mL BrdU (Sigma-Aldrich) (reconstituted in 0.007 N NaOH and 0.9% PBS). An hour later, the animals were sacrificed by cervical dislocation. Quickly, the entire digestive tract was removed on a cooled surface, and the organs were massed (Analytical Balance, AUX Series, Shimadzu Scientific Instruments). The intestine was extracted, and then the intestine contents were gently removed mechanically. The mass and length of the intestine were recorded (±0.001 g and 0.1 cm respectively). Then, the intestine was cut longitudinally and used for histological analysis. The segment chosen for histology was fixed in 4% PFA (Sigma-Aldrich) for 2 h at 4°C followed by dehydration overnight in 30% sucrose (Merck) solution. The tissues then were embedded in OCT (Tissue-Tek, United States) and placed in disposable vinyl specimen molds (Tissue-Tek Cryomold). The OCT pieces were cut into 14 μm thick slices using a cryostat.

#### Histological Analysis

The slides were washed for 5 min in PBT (PBS + 0.1% Triton (Triton^®^ x-100 surfactant, Sigma-Aldrich). Then, the slides were incubated in the dark for 5 min in a solution of methanol and 0.3% H2O2 to inhibit endogenous peroxidase activity. The samples were then washed with PBT, and an epitope-unmasking protocol was performed by immersing the samples in a 10 mM citrate/1 mM EDTA buffer solution (pH 6) for 1 h at 80°C and then for 2 min in a solution 0.1% NaBH4. In order to denature the DNA from the samples, the slides were immersed in 2 N HCl at 37°C for 30 min and neutralized with 0.1 M Na2B4O7 (pH 8.5) for 2 min. Subsequently, the slides were blocked by immersing in a serum (ABC kit; R.T.U Vectastain) for 1 h. Then they were and incubated with 1: 100 monoclonal mouse Anti-Bromodeoxyuridine Clone Bu20a (BrdU, Dako) primary antibody, Anti-Cleaved Caspase-3 (Cell Signaling) or Anti-Phospho-histone H3 (Merck Millipore) overnight at 4°C. Next, the excess primary antibody was washed, and the ABC secondary antibody kit (R.T.U Vectastain) was used according to the manufacturer’s instructions. Finally, after washing with PBT, presence of the secondary antibody was revealed by incubating in 1:10 DAB (diaminobenzidine, Roche) for 6 min. After this, the nuclei were stained with hematoxylin (Merck Millipore) (1:10) for 30 s, and the samples were dehydrated with an alcohol gradient. The samples were then maintained in xylene and mounted with Entellan medium (Merck Millipore). The microphotographs were taken at 40X and 100X with an optical microscope (Olympus BX51) equipped with a digital camera (Moticam 2500).

#### Analysis of Histological Samples

Each treatment (control and HBOT) included three individuals. For the histological analysis, 36 crypts from the first third of the intestine of each individual were randomly chosen. The number of cells that were positive for BrdU, phosphorylated histone-H3 (P-Histone 3), or Cleaved Caspase 3 per villus of each crypt were scored by two independent counters.

### Measurements of the Metabolic Rates of Chicken Embryos

**FIGURE 2:**
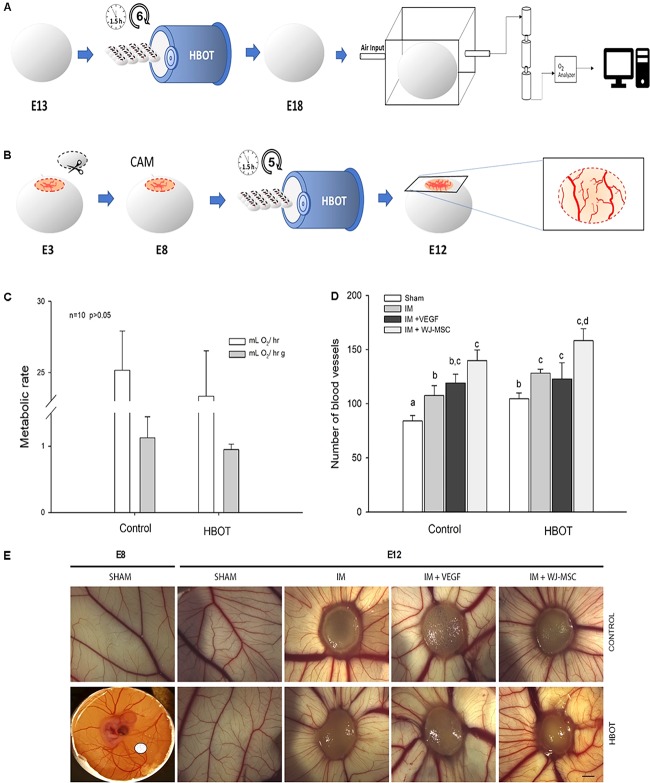
Hyperbaric oxygen therapy (HBOT) effects on chick development and angiogenesis. **(A)** Metabolic assay scheme using chicken embryos, broiler strain (Ross). Embryos were treated with HBOT from E13 to E18. Standard flow-through respirometry was used. **(B)** Schematic cartoon of the CAM assay. The CAM experiment was started at E8, and the embryos where treated until E12; the blood vessels were counted using ImageJ. VEGF-A (20 μL on IM, 10 ng/μL) was used as a positive control. **(C)** Metabolic rate of embryos in the control and HBOT groups. White bars show embryo metabolic rates (mL O_2_ h g^−1^); no differences were found between treatments. Gray bars indicate mass-specific metabolic rates (mL O_2_/h g) of the control and HBOT groups; no significant differences were found between groups. **(D)** Quantification of the number of blood vessels in the CAM after applying different stimuli. Combined use of HBOT and WJ-MSC-enriched IM scaffolds promoted a maximum of angiogenic response. **(E)** Representative images of E8 and E12 CAM after receiving treatments as indicated. Data are reported as mean ± SD. Different letters denote differences after Tukey *post hoc* tests. Bar = 5 mm.

Fertilized chicken eggs (*Gallus gallus*, Broiler strain (Ross), Agricola Chorombo, Chile) were incubated at 38.5°C and with 90% relative humidity in an incubator with circulating air (G.Q.F. MFG. Co., United States). At embryonic day 3 (E3), the eggs were briefly cleaned with 70% ethanol, and 3 mL of albumin was extracted from each egg. After this, the eggs were returned to the incubator. The eggs were randomly assigned to one of two groups, control or HBOT (12 eggs per group). The HBOT protocol is summarized in **Figure 2A**. Briefly, the eggs were exposed to HBOT conditions from E13 to E18 during six sessions. Total egg metabolic rates (embryo + extraembryonic membranes) were estimated as oxygen consumption ($\dot{V}$O_2_) when E18 eggs were subjected to standard flow-through respirometry conditions, after the last HBOT session. Four eggs were used for each treatment. The metabolic rate measurements were performed similarly to that described above for mice. But contrary to the aforementioned protocol, the eggs were placed in a transparent acrylic chamber (300 mL) located in a temperature controlled (Ta = 38.0 ± 0.5°C) dark cabinet (Sable Systems, Henderson, NV, United States). The metabolic chamber received air from a mass flow controller and through Bev-A-Line tubing after being dried at 130 mL ^∗^ min-1. Our sampling interval was 1 s with measurements performed over periods of 30 min or until a plateau in oxygen consumption levels was obtained (Peña-Villalobos et al., 2017).

### Organ Mass

After the metabolic determinations, all of the embryos analyzed were removed from the eggs and sacrificed by decapitation on a cold surface. The organs (liver, heart and gizzard) and CAM were removed and weighed (±0.0001 g).

### Chicken CAM Assay

Having established the basal physiological parameters, the HBOT and control groups were further subdivided into four treatments. The first treatment, referred to as sham, included individuals who only where subjected to egg opening. The second treatment, involved treating the animals with a commercial wound care device that provides a scaffold for cellular invasion and capillary growth, Integra Matrix Wound Dressing (IM). The third treatment, IM + Vascular endothelial growth factor (VEGF-A, Invitrogen), involved treating with a recombinant pro-angiogenic factor embedded in the scaffold. Finally, the IM + WJ-MSCs treatment included a cell-based approach consisting of 6 mm of IM seeded with 5 × 10^5^ cells.

In brief, the CAM assay protocol was as follows: At E3, a 2 cm^2^ window was opened in the egg shells. At E8, IM, IM + VEGF or IM + WJ-MSCs were placed over the CAM and an initial photo was obtained. The eggs were exposed to HBOT conditions from E8 to E12 receiving five sessions (**Figure 2B**).

The angiogenic response was digitally imaged every 24 h with an IC80 HD digital camera (Leica, Germany). At E12, commercial milk cream was added below the CAM before photographing; more details can be found in (Prieto et al., 2017). In order to quantify the number of blood vessels crossing a given area, we determined a perimeter of 10 mm for scaffolds around stimulus. For quantification, we used the software Image J (NIH).

### Wound-Healing Assay in Diabetic Mice

### Animals

We used B6.BKS(D) -Lepr db/db adult males (from now on called db/db group; for genotyping refer to **Supplementary Figure S2**) along with their wild- type (WT) siblings as our spontaneous type II diabetic animal model. Individuals (*n* = 20, 8–11 weeks) were maintained in a 12-h light/dark cycle and at a temperature of 24°C ± 2. The animals were given water and fed ad libitum with a standard laboratory diet (LabDiet, Prolab RMH 3000). At the time of surgery, the average body weights were 25.21 ± 7.96 and 31.43 ± 4.76 g in WT control and db/db mice, respectively. Blood glucose was determined using a glucometer FreeStyle Optium Neo (Abbott), being 215.00 ± 72.7 and 320.67 ± 182.4 (mg/dL) in WT control and db/db mice, respectively.

### Excisional Wound Splinting Model

**FIGURE 3:**
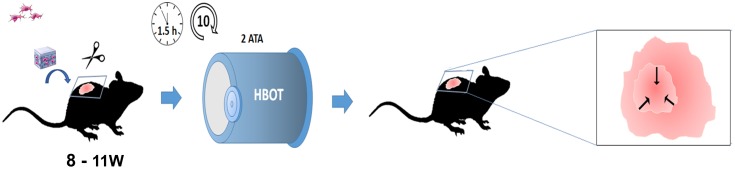
Hyperbaric oxygen therapy protocol for wound-healing in mice. Schematic representation of the wound- healing assay. Either WT or C57 diabetic adult males with excisional wound-splinting were subjected to different amounts of HBOT sessions (from 3 up to 10 sessions). The dermal reparative potential of a stem cell-seeded scaffold was tested by delivering WJ-MSC onto IM in combination with or without HBOT (IM, Integra Matrix scaffold; WJ-MSC, Wharton Jelly Mesenchymal Stem Cells). The total wound area was measured after completing the last HBOT session.

Under general anesthesia (injection with ketamine 120 mg/kg/xylazin 24 mg/kg, administrated prior to wounding), the dorsal surface of the animal was shaved, sterilely prepped, and draped for aseptic surgery. The animals were operated in sterile conditions, in the prone position, and on a heating mat (8 Watts). An excisional wound was created by using a 6 mm sterile skin biopsy punch (protocol adapted from Wang et al., 2013) (**Figure 3**). Afterward, the skin was replaced with either control or WJ-MSC-seeded scaffolds. Next, a silicone O-ring was placed surrounding the wound and attached to the skin to prevent wound-healing by contraction (**Supplementary Figure S3**). Finally, a semi-occlusive dressing (Tegaderm Film) was applied circumferentially around the trunk of the animal. Animals were monitored daily. After the last HBOT session, the animals were euthanized with anesthetic overdoses (intraperitoneal injection with ketamine 240 mg/kg/xylazin 48 mg/kg). Then, the entire skin from the animals’ backs was removed for wound area measurements (3, 5, 7, 10, and 14 days).

### Human WJ-MSC Isolation and Culture

Healthy pregnancies were considered as those where mothers were non-smokers, normotensive, had normal cholesterol levels, and did not have pre-eclampsia, pre-gestational or gestational diabetes, no family history of premature vascular diseases, and no regular consumption of medication. Independent ethics committees from the University of Chile, the Dr. Luis Tisné Brousse Hospital, and the Servicio de Salud Metropolitano Oriente (SSMO) approved the study. Written informed consent from all patients was obtained prior to enrollment in the study, and umbilical cords were obtained within 2–24 h after cesarean delivery. Standard procedures were followed for WJ-MSC isolation and characterization (Edwards et al., 2014; Prieto et al., 2017). Only established WJ-MSC cultures (passages 2–5) from different donors were used in this study.

### Statistical Analysis

All of the CAM assay determinations were carried out in at least triplicate. The data values were represented as means ± SDs. Comparisons of metabolism and organ masses between two and more groups were performed using an analysis of variance (ANOVA). Due the small sample size of diabetic animals, wound areas were compared using a permutation test (Pt) of differences based on 10,000 permutations, performed in R (R Development Core Team, 2009).

Differences in the distributions of the positive marker cells (i.e., BrdU, Cleaved Caspase 3 or P-Histone 3) in mice were analyzed with a Kolmogorov–Smirnov test with a Bonferroni correction.

All statistical analyses were performed using the STATISTICA statistical package for Windows and “R” version 3.1.2. for Windows.

## Results

### HBOT Has No Effect on Rodent Organ Masses and Metabolism

Experimental treatment had no effect on adult mouse weight or organ mass after ten sessions of HBOT (see **Table 3**). As shown in **Figure 1C**, the mass-specific resting metabolic rates (total BMR and mass-specific BMR) of individuals were statistically indistinguishable between the groups [ANOVA: *F*_(1,4)_ = 0.241, *p* = 0.649; *F*_(1,4)_ < 0.001, *p* = 0.978, respectively].

**Table 3:** Effect of HBOT on morphological traits in mice (BALB/c strain).

Organ	Control	HBOT	*F*_(1,4)_	*p*- value
Body mass (g)	26.399 ± 1.738	26.891 ± 1.555	0.134	0.733
Liver (g)	1.232 ± 0.082	1.253 ± 0.127	0.055	0.826
Intestine (g)	1.066 ± 0.044	1.131 ± 0.152	0.576	0.503
Stomach (g)	0.162 ± 0.006	0.153 ± 0.011	1.302	0.318
Lungs (g)	0.223 ± 0.065	0.199 ± 0.040	0.299	0.614

### HBOT Increases Cellular Proliferation and Turnover in Murine Intestine

Among numerous environmental factors, local available oxygen is known to regulate stem cell dynamics. We did not find a significant effect of HBOT on cell proliferation as all groups had similar average BrdU (+) cells per crypt [ANOVA: *F*_(1_,_4)_ = 4.595, *p* = 0.099]. However, the analysis of the distribution of BrdU (+) cells revealed differences between the HBOT group and the control group (Kolmogorov–Smirnov α < 0.008). While the distribution of the control group had four peaks, the distributions of mice treated with HBOT had three peaks (**Figure 1D**); specifically, 7–10, 12–14, and 15–20 BrdU (+) cells/crypt. However, we did not find differences in the distributions of cells with Cleaved Caspase 3 and P-Histone 3 (*D* = 0.181; *p* = 0.171 and *D* = 0.194; *p* = 0.460, respectively) (**Supplementary Figure S4**). These results indicate that the treatment affected the cell cycle, particularly cells in S phase, but did not affect cell death.

### HBOT Does Not Affect Chicken Embryo Physiological Parameters

We investigated the possible consequences of high oxygen tension on the metabolism of chicken embryos. We measured the effects of HBOT on embryos after 13 days of development because at this stage eggs have finished the development of the CAM (endothelial cell mitotic index declines rapidly, Ribatti et al., 2001). Moreover, the greatest increases in embryo mass occur from E12-E13 (Mortola and Al Awam, 2010). The HBOT treatment was administered until E18 in order to avoid chicken piping as other studies have shown that embryos consume more oxygen and experience dramatic changes in nutritional and metabolic state after 18 days of development (Peña-Villalobos et al., 2017). We did not find any effect of HBOT on the wet or dry mass of the E18 embryos [ANOVA: *F*_(1,18)_ = 0.264, *p* = 0.613; *F*_(1,17)_ = 1.712, *p* = 0.208, respectively]. However, we found at E18 that the masses of the legs [ANOVA: *F*_(1,17)_ = 6.411, *p* = 0.024; ANCOVA: *F*_(1,16)_ = 6.680, *p* = 0.020], liver [ANOVA: *F*_(1,17)_ = 5.672, *p* = 0.029; ANCOVA: *F*_(1,16)_ = 3.535, *p* = 0.078] and pectoral muscle [ANOVA: *F*_(1,17)_ = 4.774, *p* = 0.043; ANCOVA: *F*_(1,16)_ = 3.230, *p* = 0.091] were lower in the HBOT-treated group than in the control group. The masses of the remaining organs did not differ between groups (see **Table 4**). Additionally, the mass-specific metabolic rates (total MR and mass-specific MR) at E18 were statistically indistinguishable between groups [ANOVA: *F*_(1,18)_ = 1.855, *p* = 0.190; *F*_(1,18)_ = 3.018, *p* = 0.099, respectively; **Figure 2C**].

**Table 4:** Morphological traits of *Gallus gallus* embryos from HBOT and control groups.

Dry organ mass (g)	Control	HBOT	*F*_(1,16)_	*p*-value
Eggs	51.790 ± 4.280	50.890 ± 2.969	0.300	0.591
CAM	0.086 ± 0.013	0.096 ± 0.045	0.457	0.508
Residual yolk	15.382 ± 6.979	14.551 ± 3.743	0.099	0.757
Embryo (dry)	4.863 ± 0.584	4.485 ± 0.675	1.712	0.208
Heart	0.054 ± 0.045	0.037 ± 0.009	1.150	0.299
Liver	0.181 ± 0.034	0.143 ± 0.037	5.672	0.029^∗^
Pectoral	0.167 ± 0.023	0.143 ± 0.024	4.774	0.043^∗^
Gizzard	0.226 ± 0.085	0.173 ± 0.027	3.235	0.090
Intestine	0.079 ± 0.016	0.075 ± 0.017	0.355	0.559
Legs	0.132 ± 0.018	0.110 ± 0.020	6.680	0.019^∗^

*Asterisks denote structures analyzed with an ANCOVA (see indications in the text).*

### HBOT Combined With Cell Therapy Provides Maximal Angiogenic Response on the CAM

We conducted a CAM assay to determine if HBOT affects cells/tissues by improving neovascularization via oxidative stress. Interestingly, when all data were considered in a factorial ANOVA, we found significantly higher CAM vascularization and an increased number of blood vessels in the HBOT group compared to that found for the control group [ANOVA: *F*_(2,18)_ = 14.379, *p* < 0.001, see **Supplementary Figure S5**). Both the sham and the IM treatment resulted in more blood vessels in individuals in the HBOT versus the control group [ANOVA: *F*_(7,19)__=_ 31.731, *p* < 0.001; Tukey test: *p* = 0.040 and 0.015, respectively, **Figure 2D**]. Consistent with our previous results (Edwards et al., 2014), the angiogenic response of the IM + WJ-MSC control group is maximal among the control groups. Still, it is remarkable that the IM + WJ-MSC subjected to HBOT had on average 14% more blood vessels than that of the respective control group, revealing a trend [ANOVA: *F*_(7,19)__=_ 31.731, *p* < 0.001; Tukey test: *p* = 0.10, **Figure 2D**]. Importantly, the IM + WJ-MSC group subjected to HBOT had 1.5-fold more blood vessels than the sham control group under HBOT (95% confidence intervals: [110.474 – 98.860] versus [170.798 – 145.870] blood vessels; ANOVA: F_(7,19)__=_ 31.731, *p* < 0.001; Tukey test: *p* < 0.001, **Figure 2D**). Thus, the combination of IM+WJ-MSC plus HBOT produces a maximal angiogenic response when compared to its sham control.

### Stem Cell Therapy Combined With HBOT Promote a Faster Wound Healing in Diabetic Rodents

**FIGURE 4:**
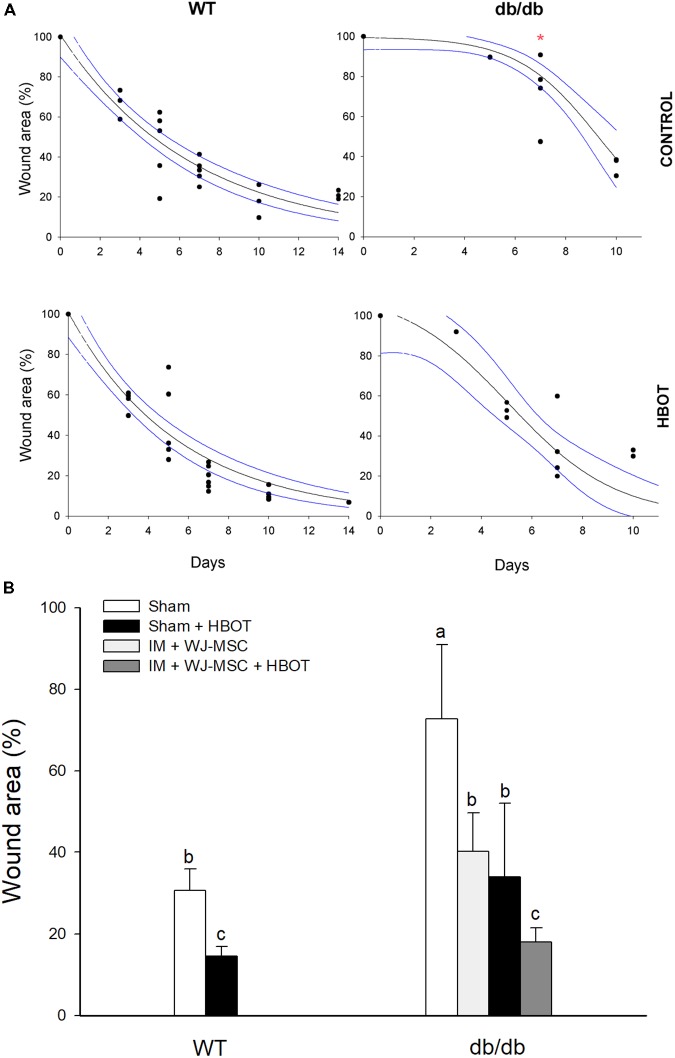
Wound-healing effects of HBOT in diabetic mice. **(A)** Wound-healing curves for the control and HBOT treated groups, including both diabetic and WT mice. Red asterisk denotes statistical differences at experimental day 7 (Pt *p* = 0.030). **(B)** Quantification of single or combined effects of HBOT and WJ-MSCs bioactivated scaffolds in diabetic mice evaluated at experimental day 7. Data are reported as mean ± SD. Different letters denote statistical differences after permutation test.

To explore whether oxygen tension impacts wound healing, we used an established diabetic genetic animal model. Although wound-healing did not differ between the WT mouse control and HBOT groups, the wound-healing time of diabetic mice that received 10 sessions of HBOT (db/db group) was less than that of diabetic mice that did not receive HBOT. On experimental day 7, wounds in db/db mice healed significantly more slower in the control group than those in HBOT treated. The wound area of db/db mice treated with HBOT was on average 50% less than that of db/db control group (72.73 ± 18.24% versus 34.02 ± 17.95%; Pt *p* = 0.03). The wound closure rate of db/db mice treated with HBOT (fit to a sigmoidal equation; 3 parameter) was comparable with the one obtained in WT mice (fit to an exponential decay; 2 parameter) modifying the healing curve and reaching comparable reduction (**Figure 4A**).

Finally, in order to evaluate the possible combined effects of cell therapy and HBOT on wound-healing, we used an IM scaffold embedded with WJ-MSCs (**Figure 4B**). At day 7, db/db animals treated with IM+WJ-MSC plus HBOT, healed significantly faster compared with db/db mice treated only with IM+WJ-MSC (Pt *p* = 0.040) or HBOT alone (Pt *p* = 0.050). In summary, our findings suggest that combining HBOT with stem cell therapy benefits wound-healing in diabetic mice.

## Discussion

### Organ Mass and Metabolism

Overall, we demonstrate that HBOT does not affect the BMR of mice (**Figure 1C**) even when the data are normalized by animal weight. Thus, energy consumption is not affected by HBOT treatment. Furthermore, we conclude that the mass-specific resting metabolic rates of the chicken embryos were statistically undistinguishable between experimental groups (control and HBOT, **Figure 2C**).

A detailed analysis of the different organ masses did not reveal differences in the mass of E18 chicken embryos between experimental groups. However, the mass of the legs was lower in the HBOT group compared to the control group. Additionally, the masses of the liver and pectoral muscle tended to be lower in the HBOT group compared to the control group when mass was corrected by egg size. These results contrast with several studies of the effect of normobaric hyperoxia, where embryo development and/or organ growth has been found to be accelerated by incubation (e.g., Smith et al., 1969; Bartels et al., 1973; Metcalfe et al., 1981; Baumann et al., 1983; Stock et al., 1983; van Golde et al., 1998). Additionally, some studies have shown that hyperoxia has minimal significant effects on pulmonary morphometry. As stated by Lewallen and Burggren (2015), the concentration of pulmonary VEGF is 30% less in HBOT E18 embryos compared to E18 control embryos. Overall, the role of VEGF in pulmonary development is not yet well understood and deserves further research.

It has been well documented that VO_2_ oxygen concentration affects metabolism (Stock et al., 1985). Despite this, after 18 days of treatment here we found no differences in the resting metabolic rates of HBOT chick embryos compared to the control group. Considering this, we propose that hyperbaric conditions could generate differential effects. Future studies should disentangle the effects of pressure and oxygen concentration during HBOT incubation.

### Cell Proliferation in the Intestine of Rodents

Oxygen availability has been known to modify the cell behavior and lineage choice of different stem cell populations *in vitro*. As the turnover rate of the tissue of the small intestine is the fastest among all mammal tissues (Snippert et al., 2010), we selected this organ as our model to study cell proliferation in response to HBOT. Hyperbaric oxygen has been used on the small intestine for the treatment and prevention of injuries such as ischemia-reperfusion (Chen et al., 1998; Daniel et al., 2011; Francis et al., 2017). In addition, HBOT has also been applied after accidents to avoid apoptosis due the lack of irrigation and oxygen (Guimarães et al., 2002); specifically, HBOT has been shown to increase the expression of VEGF (Francis et al., 2017). More recently, diseases like Chron’s syndrome have been shown to benefit from HBOT (Bosa et al., 2017). Here, we analyzed the effect of hyperbaric oxygen on the proliferation rates of the cells in Liberkühn crypts. In these crypts, intestinal stem cells and Paneth cells interact (De Mey and Freund, 2013) and make cellular turnover possible by their proliferation, differentiation, and migration through villi. Nevertheless, recently Parker et al. (2016) have shown that cellular proliferation is the principal driving force for cell migration in villi.

We did find significant differences in the distribution of BrdU (+) cells between the control and HBOT groups. Specifically, the control group had many crypts with few BrdU (+) cells (mode: 5 BrdU (+) cells) and the HBOT group had fewer crypts with more BrdU (+) cells (mode: 8 BrdU (+) cells) (**Figure 1D**). As such, it is possible that HBOT causes stem cells to proliferate in a more organized way (**Figure 1E**).

To date, no mechanism has been proposed in order to explain the changes observed in the proliferation dynamics in the small intestine following HBOT treatment. We suggest that HBOT could maximize mitochondrial ATP production by affecting many molecular pathways such as the AKT and the PI3K pathways (Sanders et al., 1966).

Of note, our experimental design did not promote changes in the length or mass of the small intestine (**Table 3**). But it is possible that HBOT could protect the individuals from intestinal injury or depletion of villi provoked by bacterial disease (Parks et al., 1982). HBOT prevents gastrointestinal damage after radiation (Bennett et al., 2005; Marshall et al., 2007). Because crypt cells respond to HBOT increasing proliferation, we suggest that these results could be related to changes in proliferation dynamics of stem cells. Future research should address whether recovery time could be shortened by this treatment.

### Hyperbaric Oxygen Induces Angiogenic Development in a CAM Assay

Since neovascularization is one of the reported beneficial effects of HBOT, we first explored this using a CAM assay. We observed increased vascularization by HBOT in both the sham and IM groups compared to the respective control non- HBOT groups (see **Figure 2D**). Despite these results, we did not find physiological differences between the groups in terms of oxygen consumption or in body mass. Based on these results, we suggest that HBOT directly modifies angiogenic development and does not modify other processes related with embryonic development, or at least embryonic development is not affected during the temporal window analyzed in this study.

To our knowledge, this work is the first to evaluate the effects of HBOT on avian vasculature development and physiology. In the literature, several studies extensively explore the effects of hypoxia and hyperoxia on CAM development. However, most of these studies (with the exception of Montecorboli et al., 2015) do not evaluate both the effects of pressure and oxygen concentration on embryonic development (Dusseau and Hutchins, 1988; Burton and Palmer, 1992; Druyan and Levi, 2012). Interestingly, there is conflicting evidence of the effect of ambient oxygen on vascular development. Burton and Palmer (1992) have reported that hypoxia diminishes embryo and CAM growth but also accelerates maturation of the capillary plexus. Additionally, these authors report that hyperoxia (in normobaric conditions) results in a thicker air-blood barrier. On the other hand, some studies (Druyan and Levi, 2012) report that the vascular area of the CAM is greater in eggs incubated at 17% atmospheric oxygen (i.e., hypoxia) compared to control group. Apparently, the response to oxygen level is highly dependent on the developmental stage of the embryo. Indeed, Chan and Burggren (2005) have shown that CAM mass is unchanged by hypoxia experienced during early or mid-development, but CAM mass at E18 is greatly increased by hypoxia. Despite these contrasting results, the present study is in agreement with that presented by Montecorboli et al. (2015) where significantly higher CAM vascularization and an increased number of blood vessels were found for E6 and E7 subjected to HBOT (protocol: 2.0 ATA, for 30 min).

The observed effects of HBOT on the CAM are similar to others reported for hyperoxic-normobaric conditions. A feasible explanation for the results obtained here could be that the CAM is responsive to decreased O_2_ concentration and normobaric conditions, and the reduced response threshold could be due to the low PO_2_ needed during embryonic development (Meuer et al., 1992).

### HBOT and IM Produce Similar Responses in CAM Assay

The results presented here provide the first evidence that the effects of HBOT on angiogenesis are similar to those of commercially available wound dressing scaffolds. Interestingly, angiogenesis has been shown to be improved when IM is embedded with WJ-MSC (Edwards et al., 2014). Despite this, we found no difference in the effects of HBOT alone or combined with WJ-MSC seeded scaffolds, IM, or the canonical angiogenic factor VEGF loaded IM (**Figure 2D**). These results suggest that HBOT has the same effects on CAM angiogenesis as do other pro-angiogenic treatments (**Figure 2E**), and as such could be an effective non-invasive treatment. Nevertheless, we would like to highlight that a cellularized scaffold subjected to HBOT still reveals a tendency in promoting more angiogenesis. Most likely, by combining IM+WJ-MSC plus HBOT we are able to induce experimentally a maximal angiogenic response, reaching a plateau around 150 blood vessels. We consider that the impossibility of developing more vessels could respond to the biological upper limit to this morphological trait (for a discussion see Parsons-Wingerter et al., 1998).

### Wound Assay in Diabetic Rodents

For the past two decades, HBOT has been used in wound-repair mostly for the treatment of foot ulcers associated with diabetes (Mason et al., 1999). In line with these results, we present evidence that the wound-healing time was less when diabetic individuals were subjected to HBOT than when no treatment was given (**Figure 4A**). Furthermore, diabetic mice subjected to the combined-treatment improved faster than those under other treatments. Specifically, the wounds of the diabetic mice subjected to HBOT plus WJ-MSC seeded scaffolds recovered significantly faster and with fewer sessions than the sham control group (**Figure 4B**). Most likely, HBOT increased the tissue oxygen content and improved collagen synthesis, angiogenesis, and epithelization in the wound bed. This being said, the use of HBOT in combination with other bioengineered scaffolds should be carefully considered. Indeed, Kim et al. (2007) have analyzed a combination of factors associated with wound healing (e.g., commercial wound care devices) and have shown that the combining HBOT with other treatments is limited by whether the substance is a potential fuel source for combustion.

During the preparation of this manuscript, another study testing HBOT on diabetic rats (induced by STZ) was published Montecorboli et al. (2015). There the authors show that the wound-healing curves of an HBOT and control group differ at day 20. Despite the fact that the hyperbaric oxygen treatment of that study differs from the one used here (2.4 ATA, 5 days a week for 6 weeks), our results overall are in accordance with their outcome. Of note, our results indicate that the healing time of the HBOT diabetic mice at day 7 already was shorter compared with the control diabetic group.

### Mechanism of Action for HBOT in Tissue Repair

Our study demonstrates that HBOT treatment modifies several aspects of tissue regeneration and repair. Specifically, HBOT increased CAM development in chicken embryos and the wound-repair capacity in a diabetic murine model.

Regarding the mechanism by which HBOT could benefit tissue repair, reactive oxygen species (ROS) and reactive nitrogen species (RNS) waves have been postulated to mediate the effects of wound-healing (Fosen and Thom, 2014). Transiently increased ROS and RNS could serve as signaling molecules, since both pathways are activated by a repeated HBOT exposure (Thom, 2009; Poff et al., 2017). In fact, numerous studies have demonstrated that HBOT can eject a beneficial effect by balancing the oxygen free radicals. Recently, hypoxia-inducible factor 1 α (HIF-1 α) has been shown to be activated (Sunkari et al., 2015) and endothelial progenitor cells have been shown to be mobilized by hyperoxia (Gallagher et al., 2007). The so-called “Hyperoxia-Hypoxia paradox” results from a balance between the positive and negative effects of the ROS/RNS and oxygen levels. The main effect is caused by repeated HBOT sessions that elevate partial pressure of oxygen leading to an increase in ROS levels, which in turn activates the endogenous anti-oxidant defense system. Hence, by increasing and then decreasing back to normal the oxygen concentration/exposure many of the mediators induced during hypoxia are being induced by hyperoxia through a negative feedback loop. For example, HIF-1 α is induced after hyperoxic exposure. Once HIF is induced it modulates other factors that promote angiogenesis such as VEGF (Hu et al., 2014).

In summary, this study underscores the importance of oxygen tension as a regulator of stem cell biology in homeostasis and disease. We conclude that oxygenation might provide an effective adjuvant aid to diverse clinical treatments.

## Author Contributions

VP and IP-V: conception and design. CPr, CPi, IP-V, IC-M, and PL: collection and or assembly of data. IP-V, IC-M, and VP: data analysis and interpretation. JL and GO: Provision of study material. IP-V, IC-M, and VP: manuscript writing.

## Conflict of Interest Statement

GO was employed by company Osorio Hermanos & Cia. Ltd. The remaining authors declare that the research was conducted in the absence of any commercial or financial relationships that could be construed as a potential conflict of interest.

## Supplementary Material

FIGURE S1 Images of the HBOT experimental chamber. **(A)** Frontal view of the chamber showing the control panel where pressure, O_2_, N_2_, and air level can be regulated. The led screen indicates the concentration of O_2_ and CO_2_. **(B)** Upper view of the chamber indicating the unit where the animals are placed. Also shown are the lateral and upper pressurized windows, an HD camera, a led light, and an analog manometer.

FIGURE S2 Representative image of a genotyping gel. Asterisks represent identification of - *B6.BKS(D) -Lepr* ^db/db^ animals (spontaneous type II diabetic model).

FIGURE S3 Representative picture of the excisional wound splinting in mice. Either WT or spontaneous type II diabetic adult C57 males were used. Shown is the 8 mm wide silicone patch with an internal hole of 6 mm to allow for air exchange.

FIGURE S4 Analysis of the distributions of P-Histone 3 (+) and Cleaved Caspase 3 (+) cells. No significant differences were found between the HBOT and control groups (Kolmogorov–Smirnov α < 0.05).

FIGURE S5 Quantification of the angiogenic effect of HBOT versus control in the CAM assay at E12.
