# Supplementary figures and images for: Hyperbaric Oxygen Increases Stem Cell Proliferation, Angiogenesis and Wound-Healing Ability of WJ-MSCs in Diabetic Mice
Source: Front Physiol. 2018 Jul 30;9:995. doi: 10.3389/fphys.2018.00995 (PMC6078002; doi:10.3389/fphys.2018.00995)

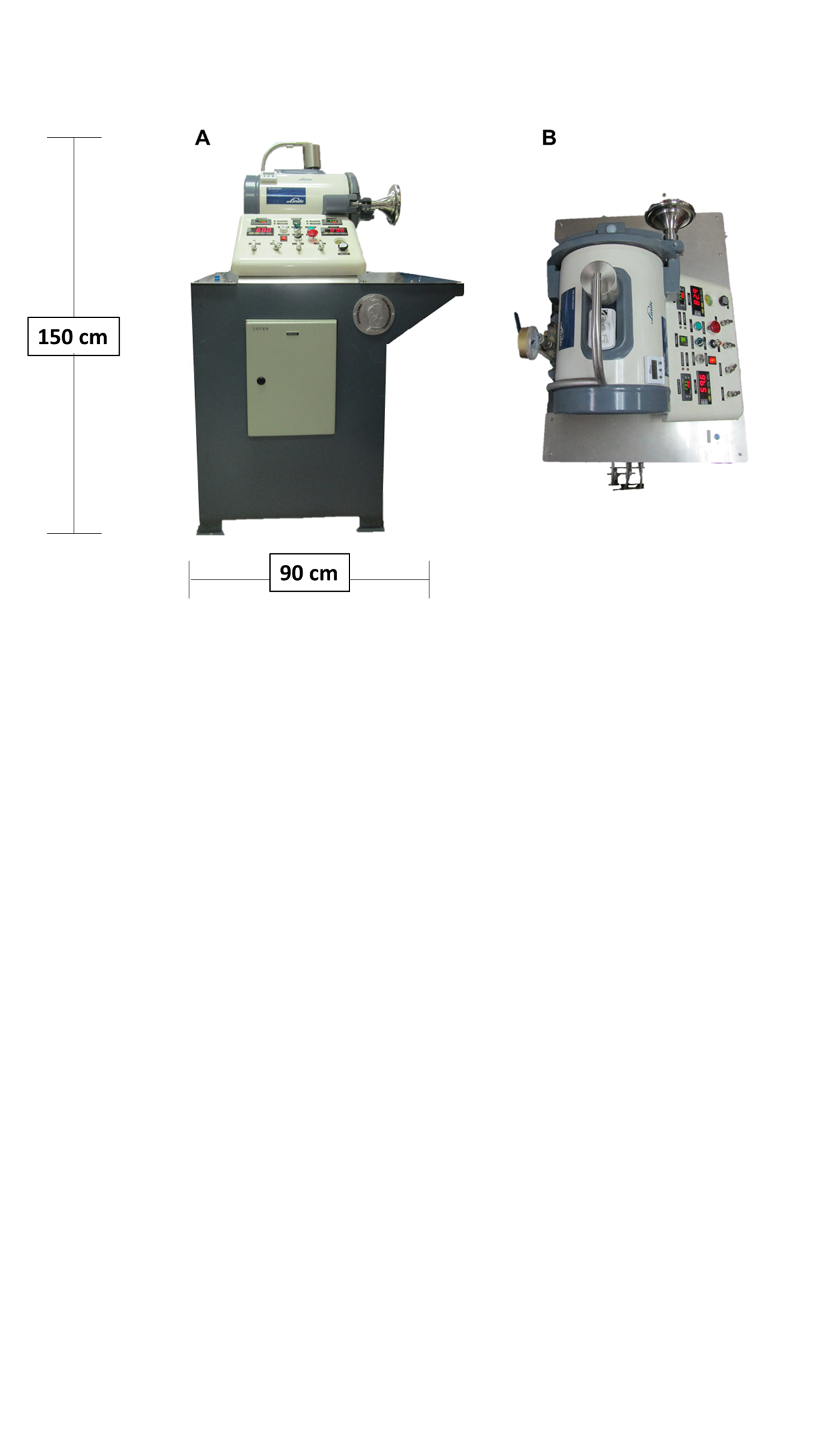

Supplement: FIGURE S1 — Images of the HBOT experimental chamber. (A) Frontal view of the chamber showing the control panel where pressure, O2, N2, and air level can be regulated. The led screen indicates the concentration of O2 and CO2. (B) Upper view of the chamber indicating the unit where the animals are placed. Also shown are the lateral and upper pressurized windows, an HD camera, a led light, and an analog manometer. [file Image_1.TIF]

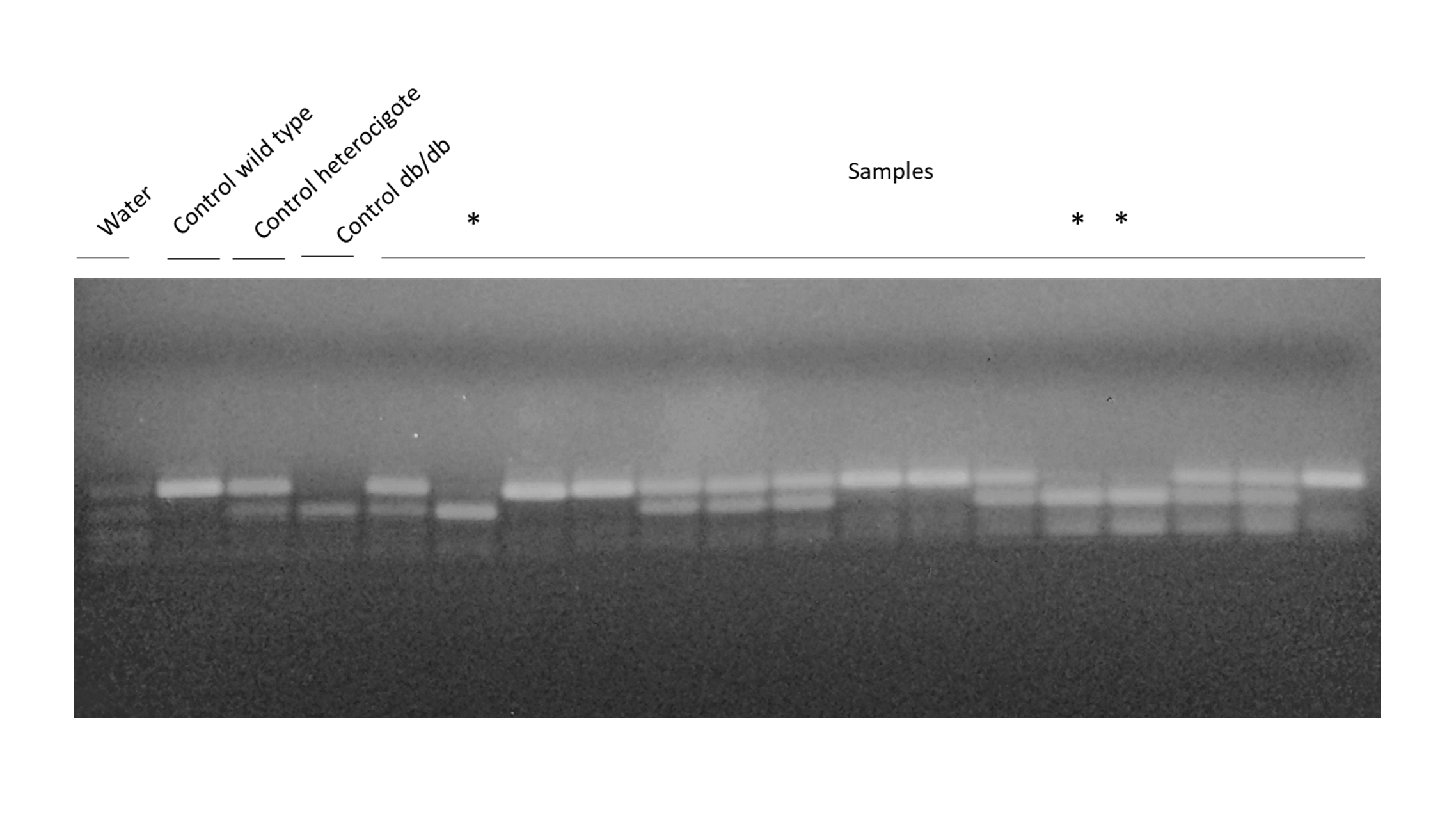

Supplement: FIGURE S2 — Representative image of a genotyping gel. Asterisks represent identification of - B6.BKS(D) -Lepr db/db animals (spontaneous type II diabetic model). [file Image_2.TIF]

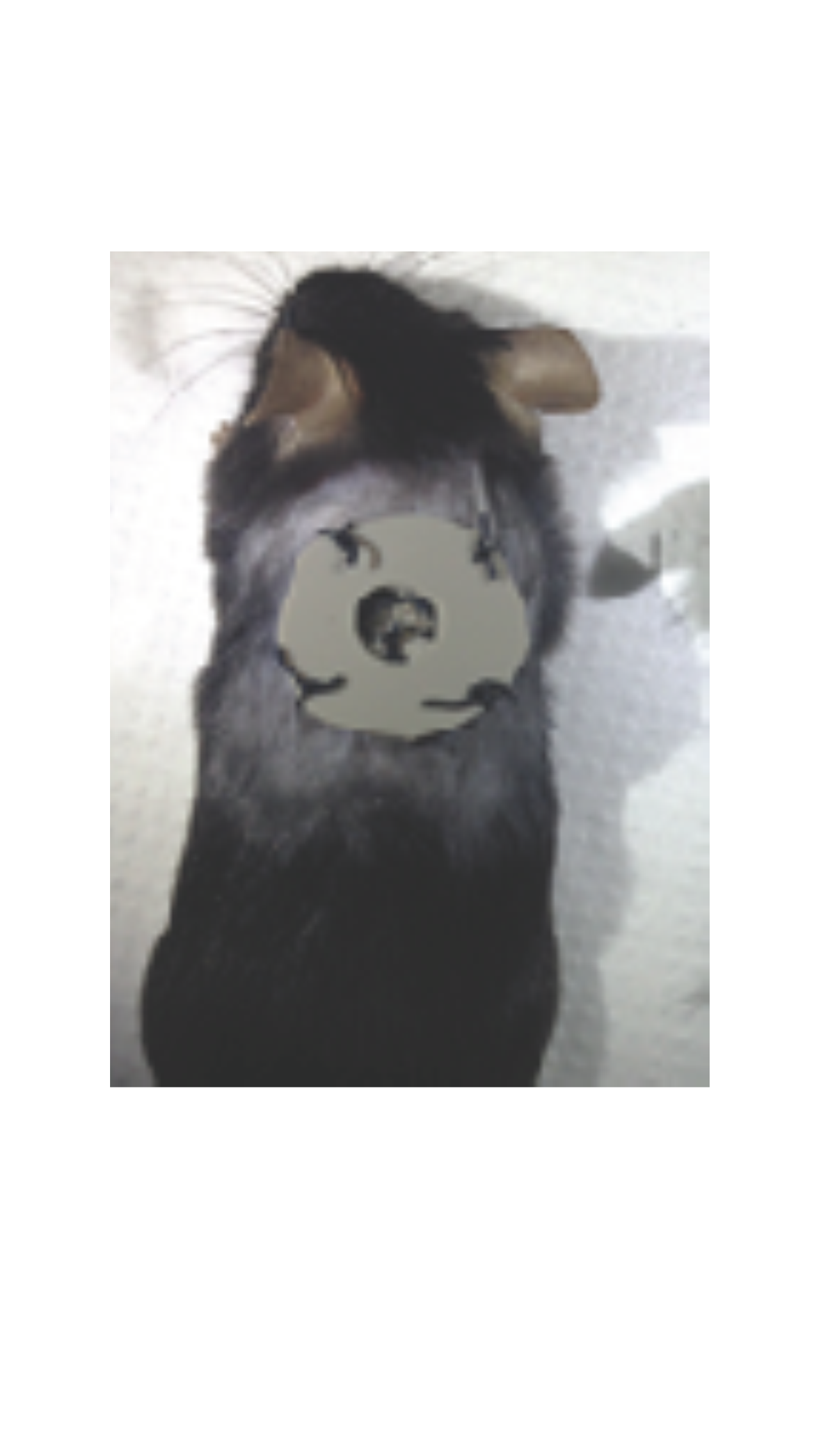

Supplement: FIGURE S3 — Representative picture of the excisional wound splinting in mice. Either WT or spontaneous type II diabetic adult C57 males were used. Shown is the 8 mm wide silicone patch with an internal hole of 6 mm to allow for air exchange. [file Image_3.TIF]

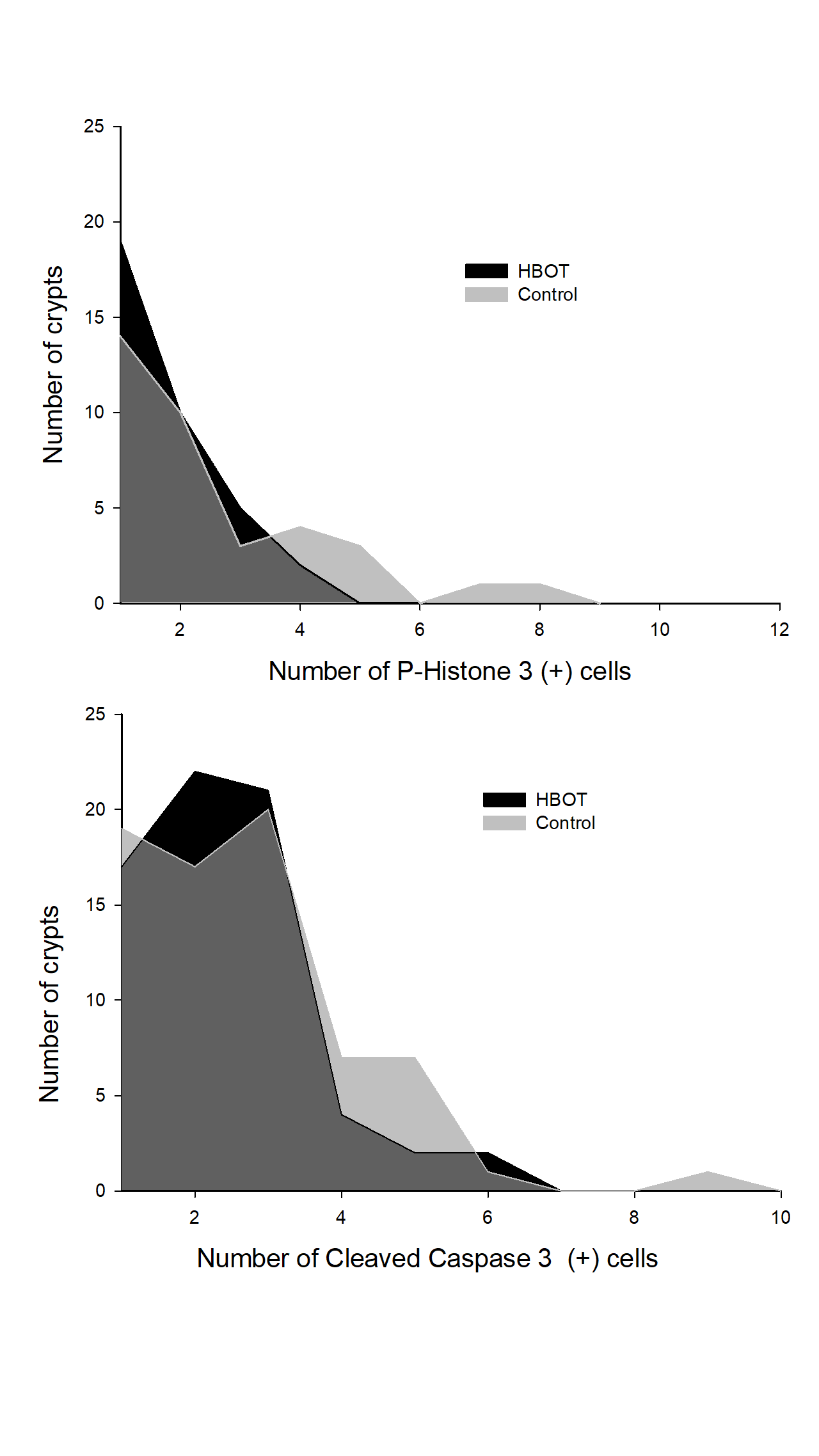

Supplement: FIGURE S4 — Analysis of the distributions of P-Histone 3 (+) and Cleaved Caspase 3 (+) cells. No significant differences were found between the HBOT and control groups (Kolmogorov–Smirnov α < 0.05). [file Image_4.TIF]

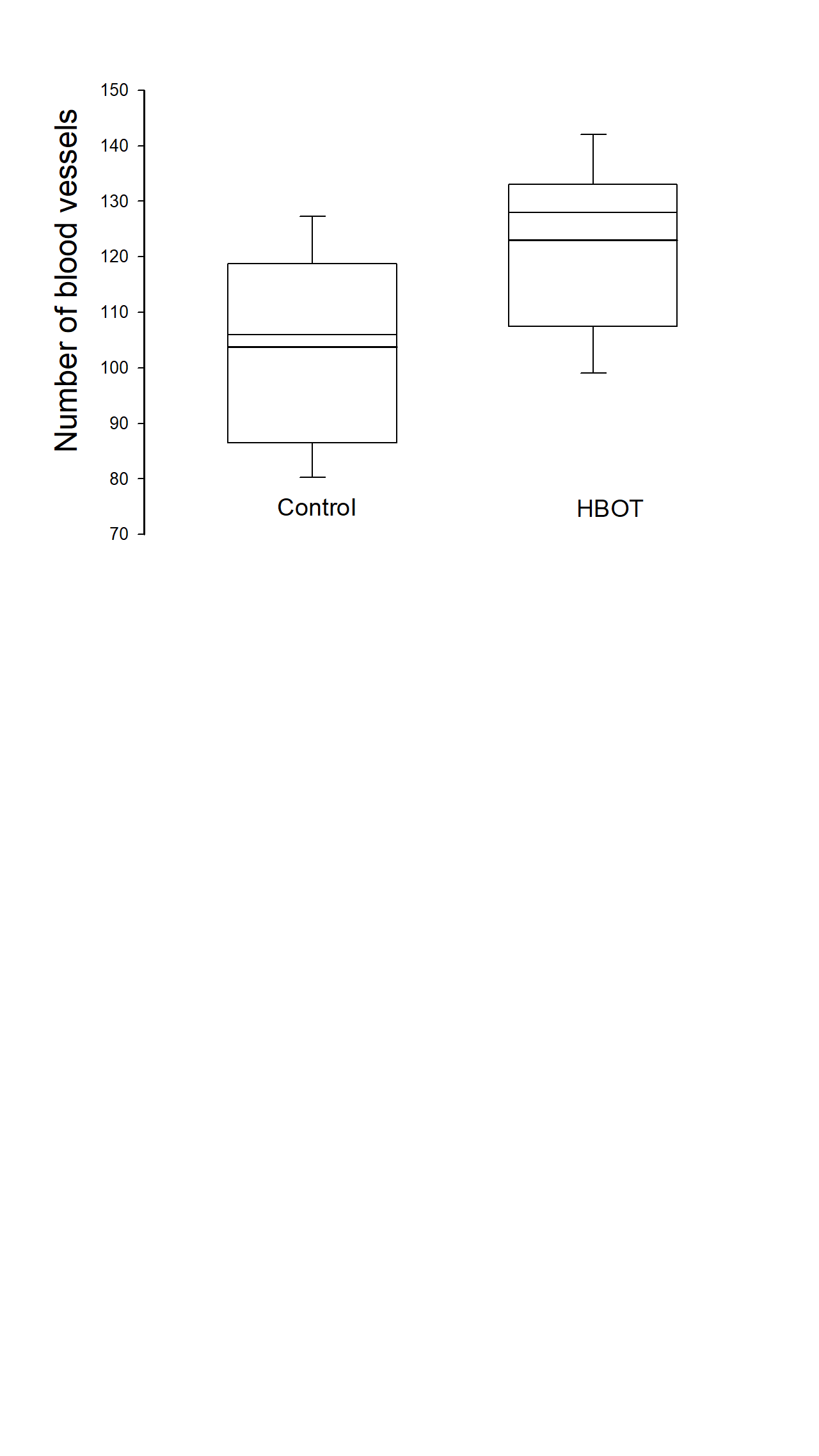

Supplement: FIGURE S5 — Quantification of the angiogenic effect of HBOT versus control in the CAM assay at E12. [file Image_5.TIF]
